# Quantity as a Fish Views It: Behavior and Neurobiology

**DOI:** 10.3389/fnana.2022.943504

**Published:** 2022-07-14

**Authors:** Andrea Messina, Davide Potrich, Matilde Perrino, Eva Sheardown, Maria Elena Miletto Petrazzini, Peter Luu, Anna Nadtochiy, Thai V. Truong, Valeria Anna Sovrano, Scott E. Fraser, Caroline H. Brennan, Giorgio Vallortigara

**Affiliations:** ^1^Centre for Mind/Brain Sciences, University of Trento, Rovereto, Italy; ^2^Centre for Developmental Neurobiology, Institute of Psychiatry, Psychology and Neuroscience, New Hunt’s House, Kings College London, London, United Kingdom; ^3^Department of General Psychology, University of Padova, Padua, Italy; ^4^Michelson Center for Convergent Bioscience, University of Southern California, Los Angeles, CA, United States; ^5^Department of Psychology and Cognitive Science, University of Trento, Rovereto, Italy; ^6^School of Biological and Behavioral Sciences, Queen Mary University of London, London, United Kingdom

**Keywords:** quantity discrimination, zebrafish, retina, tectum, visual system, pallium, fish cognition, imaging

## Abstract

An ability to estimate quantities, such as the number of conspecifics or the size of a predator, has been reported in vertebrates. Fish, in particular zebrafish, may be instrumental in advancing the understanding of magnitude cognition. We review here the behavioral studies that have described the ecological relevance of quantity estimation in fish and the current status of the research aimed at investigating the neurobiological bases of these abilities. By combining behavioral methods with molecular genetics and calcium imaging, the involvement of the retina and the optic tectum has been documented for the estimation of continuous quantities in the larval and adult zebrafish brain, and the contributions of the thalamus and the dorsal-central pallium for discrete magnitude estimation in the adult zebrafish brain. Evidence for basic circuitry can now be complemented and extended to research that make use of transgenic lines to deepen our understanding of quantity cognition at genetic and molecular levels.

## Quantities and Their Ecological Importance in Fish Behavior

Quantity discrimination is a fundamental aspect of our everyday life. A preference for more or less of something can be easily observed in non-human animals’ behavior. Extensive evidence in several species supports the use of quantity estimation during foraging (e.g., Hauser et al., 2000; Bar-Shai et al., 2011; Garland et al., 2012; Stancher et al., 2015; Yang and Chiao, 2016; Gazzola et al., 2018), defensive responses (e.g., McComb et al., 1994; Wilson et al., 2002), reproductive and safety strategies (e.g., Queiroz and Magurran, 2005; Carazo et al., 2009; Rooke et al., 2020), and parental cares (e.g., Lyon, 2003). Indeed, the ability to estimate and process the number of elements in a group or the size of another animal (prey or predator) is widespread across different species, which suggests it may be of highly adaptive value in their ecological niches (see reviews in Vallortigara, 2017; Nieder, 2020; Bortot et al., 2021; Messina et al., 2021b).

In particular, fish have mainly been studied by taking advantage of their social responses. Fish form shoals (i.e., aggregation of conspecifics) to dilute the risk of being preyed upon (Foster and Treherne, 1981). Quantity discrimination can, thus, be studied by giving fish the possibility to join either one of two groups of companions differing in number.

For example, angelfish reliably choose the larger set of conspecifics in 1 vs. 2 and 2 vs. 3 comparisons but is at chance in 3 vs. 4 comparisons (Gómez-Laplaza and Gerlai, 2011a). Mosquitofish show more accurate performances, discriminating 3 vs. 4 but not 4 vs. 5 (Agrillo et al., 2008), whereas guppies can discriminate with this ratio (Lucon-Xiccato et al., 2017). These results indicate that fish show a discriminatory limit of 3–4 elements among small numerosity, as found in other vertebrate species (e.g., Stancher et al., 2015). Fish, however, also proved to be able to compare sets with large numerosity (>4 elements), showing a ratio-dependent accuracy. Angelfish can discriminate up to a 0.56 ratio (e.g., 5 vs. 9; Gómez-Laplaza and Gerlai, 2011b), while other species such as guppies (Agrillo et al., 2012), mosquitofish (Agrillo et al., 2008), and swordtail (Buckingham et al., 2007) show a limit set at 0.5 (one group is two times the other). Fewer evidence indicates that fish can go higher than 0.67: three-spined sticklebacks discriminate up to 0.87, showing, however, a progressive accuracy decrease as the ratio increases (Mehlis et al., 2015). The size of the shoal matters not only to prey but also to predators. Many fish species suffer a low capture success when attacking groups of prey, due to known anti-predator benefits of grouping such as predator confusion (Milinski, 1977a,b). For example, white perch direct more attacks at stragglers than shoals of killifish (Morgan and Godin, 1985). However, an opposite trend has been found in acaras and pike cichlids that showed to direct their attack toward the larger shoal of prey (Krause and Godin, 1995; Botham et al., 2005).

The discrimination of food quantities is one of the most relevant abilities from an ecological point of view. As stated by the theory of optimal foraging, a larger food patch offers a higher energetic gain (Krebs et al., 1974). Guppies spontaneously select the larger number of food items with contrasts of 1 vs. 4 and 2 vs. 4 (ratio 0.5, Lucon-Xiccato et al., 2015). Similarly, angelfish discriminate between identically sized food quantities with a numerical ratio of up to 0.67 (Gómez-Laplaza et al., 2018).

Quantity discrimination has also been described in fish concerning parental care: females of convict cichlid spend more time trying to recover fry from the larger of two groups displaced from the nest, by up to a 0.67 ratio (6 vs. 9, Forsatkar et al., 2016). In mating strategy, females of mouthbrooder cichlid prefer males that show more spots in their tails mimicking the conspecific eggs (Hert, 1991).

Studies in fish investigating relative quantity judgments revealed that their accuracy parallels that of many other vertebrates: with small numerosity, discriminative accuracy stands at around 3–4 elements, but when numerosity increases, the discrimination appears to be ratio-dependent, following Weber’s Law (accuracy decreases as the ratio increases, Cantlon and Brannon, 2006).

## The Issue of the Control of Continuous and Discrete Quantities in Behavioral Studies

All the studies mentioned in the previous paragraph face a drawback: the discrimination of relevant ecological stimuli and their numerosity is intertwined with other non-numerical continuous magnitudes. For example, when the number of conspecifics or food items increases, other aspects would, most likely, also increase: e.g., the overall volume, area, and perimeter tend to correlate positively with numerical information. Similarly, if the elements are equally spaced, the larger group will globally occupy a larger space (also known as convex hull or sparsity); a balance of the convex hull, on the other hand, leads to having different items’ density. Also, the continuous and discrete aspects of the quantitative features of a stimulus can interact with one another (review in Vallortigara et al., 2022). The type of information the animals pay attention to during discrimination is a challenge for this kind of research.

In food quantity discrimination tasks, both angelfish (Gómez-Laplaza et al., 2019) and guppies (Lucon-Xiccato et al., 2015) were found to prefer larger-sized food items as opposed to larger food amounts. As to the control of items’ spatial disposition, when the inter-item distance was kept constant, angelfish preferred the more numerous to the less numerous set (Gómez-Laplaza et al., 2018). However, the density of the food elements appeared to be an important feature for angelfish when dealing with large numbers (e.g., 5 vs. 10), driving the choice toward the smaller sets with clustered (denser) items concerning the numerically larger sets with scattered items. The density of the group’s items seems to be particularly relevant for fish, as well as in social contexts, affecting shoal discrimination in angelfish (Gómez-Laplaza and Gerlai, 2013) and three-spined stickleback (Frommen et al., 2009).

The role of swimming activity (and, thus, the amount of stimulus motion) is another important non-numerical cue, being usually higher in a larger group. One method to equalize this continuous variable between the sets is to reduce the water temperature (as the temperature decreases, fish activity decreases) or to restrict the space occupied by each fish in the stimulus shoal. When the temperature was manipulated, a loss of preference for the larger shoal was described in zebrafish (Pritchard et al., 2001). Similarly, mosquitofish and angelfish shoaling discrimination was affected in large comparisons (respectively, 4 vs. 8 and 5 vs. 10) but not in small comparisons (2 vs. 3; Agrillo et al., 2008; Gómez-Laplaza and Gerlai, 2012). However, when activity was controlled by space restriction, angelfish preferred the larger shoal in both numerical contrasts (Gómez-Laplaza and Gerlai, 2012).

As to the area of the stimuli, in mosquitofish, this has been controlled for by placing larger individuals in the numerically smaller stimulus shoal and smaller individuals in the numerically larger stimulus shoal. In such a condition, fish showed no significant choice preference, highlighting the relevance of the overall stimuli area and/or the individual size of the stimuli in discrimination (Agrillo et al., 2008). Similar results were recently obtained in guppies, zebrafish, Chinese crucian carps, and qingbo (Xiong et al., 2018).

Another technique to prevent fish from using continuous quantities involves reducing the visual access to the conspecifics by partial occlusion. For example, fish can initially observe two different numerical shoals simultaneously; then, before allowing individuals to exhibit a preference between the two sets, some individuals from the larger group are occluded, leaving the same number of the stimuli visible in the two shoals. In this condition, redtail splitfins chose the larger shoal in small numerical comparisons (1 vs. 2, 2 vs. 3, but not 3 vs. 4, Stancher et al., 2013). Similar performances with both small and large numerosity were obtained in zebrafish (Potrich et al., 2015) and angelfish (Gómez-Laplaza and Gerlai, 2015), although the latter were less accurate with large numerosity. Using an adaptation of the “item-by-item presentation” procedure, mosquitofish were exposed to a shoal test task, in which each fish stimulus was singly confined in separate compartments, with several opaque occluders positioned in such a way that the test subject could see only one stimulus at a time. Fish were, therefore, required to add the amount of the seen conspecifics on both sides and compare them. Mosquitofish spent more time close to the largest shoal in 2 vs. 3 and 4 vs. 8 comparisons (Dadda et al., 2009).

The importance of non-numerical versus numerical information in quantity discrimination may have ecological reasons. For example, while foraging, it may be more relevant for fish to select the larger-sized food items instead of the larger numerosity, motivated by the attempt to maximize energy gained from eating the food while minimizing energy expenditure collecting and/or protecting the food. Similarly, the density of conspecifics in the shoal could sometimes be more relevant than pure numerical information to gain protection in the group. Hence, the use of experimental procedures based on spontaneous preferences for attractive ecological stimuli prevents us from studying the role of different quantities (number, size, density, etc.). The use of conditioning procedures might overcome some of these limitations.

During conditioning procedures, animals are typically requested to discriminate between different sets of elements differing in numerosity by choosing the one associated with a reward (usually food or a social reward). Using this method, mosquitofish were trained to discriminate between different sets of two-dimensional stimuli (2 vs. 3) to gain access to social companions (Agrillo et al., 2009). Fish discriminated between the two numerosity when no control on the continuous stimulus variables was done but showed a drop in performance when tested after equalizing some of the continuous quantities. For instance, when the cumulative surface area or the convex hull (sparsity) were equalized between the groups, performance dropped to chance level and no interference was found when overall perimeter or total brightness were balanced. Similar results were obtained in the same species when large numerosity was used (4 vs. 8 and 100 vs. 200, Agrillo et al., 2010). Using a different technique, cavefish showed to discriminate between different quantities of vertical sticks associated with a food reward only when both continuous quantities were correlating with numbers but not when only numerical information was available (Bisazza et al., 2014a).

The lack of numerical performance found in these studies might outline a fish’s inability to use numerical cues and/or the fact that numerical information could be taken into account by fish only when other non-numerical information (such as overall area or convex hull) is not available (as a last resort strategy). Interestingly, however, when trained with controls for non-numerical cues from the beginning of the training, mosquitofish and cavefish showed that they can discriminate based on numerical information alone (Agrillo et al., 2010; Bisazza et al., 2014a). Moreover, the use of numerical cues alone can be conducive to high accuracy levels (higher than 90%), provided fish are exposed to extensive training, as shown in guppies (Bisazza et al., 2014a) and goldfish (DeLong et al., 2017). Furthermore, the hypothesis that relying on numbers would represent a last resort strategy was tested by Agrillo et al. (2011) who trained mosquitofish to discriminate between two sets of items making available either only continuous variables, only numerical information, or both simultaneously. As expected, fish learned to discriminate more quickly when both numbers and continuous information were available than when only continuous information or only numerical information could be used. Interestingly, there was no difference in learning between the two latter conditions, suggesting that the process of learning numbers is no more cognitively demanding than that of learning continuous variables (Agrillo et al., 2011).

The abovementioned studies in fish attempting to control for non-numerical variables during the learning process were mainly focused on the overall elements’ area, density, and convex hull (see e.g., Agrillo et al., 2012; Bisazza et al., 2014b; DeLong et al., 2017). In a recent study, archerfish were trained to select one of two groups of items (small dots) (3 vs. 6 and 2 vs. 3 elements), with accurate control for all the possible geometrical constraints and their combinations (elements’ size, overall area, overall perimeter, density, and sparsity), ensuring that only numerical information was available (Potrich et al., 2022). Results confirmed that non-numerical cues (including the spatial frequency of the stimuli used) did not correlate with the archerfish’s performance accuracy, suggesting that the discrimination made by the animals was based on purely numerical information.

## Quantitative Abilities in Zebrafish

Despite the huge literature on fish quantitative abilities, all the studies mentioned so far are limited to behavioral observations as none of the studied species is a model in research fields such as genetics, neurobiology, and neuroimaging.

Concerning this issue, during the last decade, zebrafish (*Danio rerio*) have gained more attention as a new powerful model for studying quantitative skills at different levels of complexity, from gene to behavior. Since its innovative use as a model organism in genetics and neurodevelopmental biology by George Streisinger in the 1960s (Walker and Streisinger, 1983), the zebrafish have rapidly become an established model for translational neuroscience (Michael Stewart and Kalueff, 2012; Brock et al., 2017; Fontana et al., 2018) due to multiple advantages that surpass those in fruit flies and rodents and that make the zebrafish an excellent compromise between system complexity and practicality (McCammon and Sive, 2015; Gerlai, 2020).

Zebrafish are physiologically homologous to mammals and possess all major neurotransmitters, hormones, and receptors (Panula et al., 2006; Alsop and Vijayan, 2009). The high degree of protein and genetic homology with humans (Howe et al., 2013), coupled with refined gene-editing tools and behavioral paradigms, make this species a vertebrate system amenable to large-scale forward genetic analyses (Wolman et al., 2011; Kalueff et al., 2013; Sheardown et al., 2022). Transparency of embryos and larvae enables *in vivo* functional imaging of neural activity and establishes the zebrafish as a powerful optogenetic tool. Other advantages include cost/space efficiency and high fecundity (laying up to 200 eggs a day), which allows high-throughput screening and rapid development, with completed organogenesis by 5 days post fertilization (dpf) (Kimmel et al., 1995; Brand et al., 2002). Of relevance to this review, zebrafish possess an elaborate behavioral repertoire and complex cognitive abilities present in higher vertebrates, including learning (associative, non-associative, spatial and aversive learning; Gerlai, 2016), long-term and short-term memory (Michael Stewart and Kalueff, 2012), and of course, quantitative skills.

It is worth noting that one of the pivotal studies on quantitative abilities in fish was conducted by Pritchard et al. (2001) who showed that zebrafish preferred the larger of two shoals (2 vs. 4) when the water temperature was kept the same. However, this preference was lost when swimming activity in the larger shoal was reduced by cooling the water, thus providing the first evidence of the continuous quantities’ influence on discriminative abilities in fish.

Since then, binary choice tests, operant training procedures, and habituation-dishabituation protocols have been adopted to further investigate zebrafish skills and to disentangle the relative salience of numerical and non-numerical information.

Female zebrafish, tested in a shoal choice task with no control for continuous quantities, showed a preference for the larger group of social companions for ratios above or equaling 0,5 (0 vs. 4, 1 vs. 4, 2 vs. 8, 2 vs. 6, and 3 vs. 6). Interestingly, as performance broke down in a 4 vs. 8 contrast, the authors suggested that having more than four fish in a shoal did not confer selective advantage as both groups provided sufficient protection from potential predators, thus leading zebrafish to treat both shoals equally (Seguin and Gerlai, 2017). As mentioned above, when Xiong et al. (2018) investigated the role played by the cumulative body surface area in shoal selection, they found that different species, zebrafish included, relied on non-numerical information rather than on number when choosing between 2 and 3 conspecifics, thus confirming the salience of non-numerical information in this type of task.

To prevent zebrafish from using continuous quantities, Potrich et al. (2015) adopted the procedure previously used with redtails splitfin, in which two numerically different shoals were partially occluded at the time of choice (Stancher et al., 2013). Male zebrafish tested with this approach proved to be able to select the larger between two shoals of females when both small (1 vs. 2 and 2 vs. 3, but not 3 vs. 4) and large numbers of conspecifics (4 vs. 6, 4 vs. 8 but not 6 vs. 8) were presented, thus providing the first evidence of zebrafish ability to rely on working memory to discriminate among quantities. Furthermore, accuracy was ratio-dependent (US) as reported in several animal species. It is known that the performance in a quantity discrimination task can be affected by the experimental procedure used, even within the same species (Agrillo and Dadda, 2007; Lucon-Xiccato and Bisazza, 2017). Methodological differences (sexual vs. social context, 2 vs. 30 trials for each subject and 23 vs 50-cm-long tank) could then explain the discrepancy observed in the 4 vs. 8 contrast compared to results obtained by Seguin and Gerlai (2017).

If on the one hand, the numerical ability has been thoroughly investigated in several species (reviewed in Agrillo et al., 2017; Agrillo and Miletto Petrazzini, 2021; Messina et al., 2021b), on the other hand estimation of continuous quantities has received relatively less attention. Recently, a novel method has been developed to quickly assess size estimation ability in zebrafish by exploiting their natural preference for passing through the larger of two holes to move in their environment. Adult zebrafish showed impressive quantitative abilities in being able to discriminate up to a ratio of 0.91 with their performance decreasing while increasing the ratio between the smaller and the larger holes (Santacà et al., 2020). Importantly, the experimental procedure has been shown to have good retest reliability and to be unaffected by the experience, which is fundamental in studies testing drug effects on diseases causing cognitive deficits or investigating the cognitive decline in normal and pathological aging.

Surprisingly, the same accuracy was observed in larvae tested at 21 dpf, whereas larvae at 7 and 14 dpf discriminated up to a ratio of 0.86 (Santacà et al., 2021). Altogether, these results suggest a limited role of maturation and experience on zebrafish’s ability to estimate areas that parallel the existence of tectal neurons by selectively responding to item size already during the first week of life (see paragraph below for more details). This high accuracy seems to be in contrast with performance observed in juvenile zebrafish tested in a spontaneous choice test to investigate the ontogeny of numerical competence (Sheardown et al., 2022). In this study, the authors adapted the procedure previously used to investigate shoal choice discrimination in angelfish, in which the stimulus fish were located in individual compartments that limited movement and avoided fish hiding each other. The results showed that fish from 31 dpf already chose the larger group in 1 vs 3, 2 vs. 5, and 2 vs. 3, but not 2 vs. 4 contrasts. In a control experiment, fish proved unable to discriminate between the shoals (2 vs. 3, 2 vs. 4, and 2 vs. 5) when the overall space occupied by the social companions was the same. However, they still chose the larger group in 2 vs. 5 and 2 vs. 3 but not 2 vs. 4 when the overall space was not controlled for. Furthermore, when the same ratio of 0.5 was maintained but the numerousness of conspecifics was changed, zebrafish discriminated between 1 vs. 2 and 3 vs. 6 individuals, failing again in 2 vs. 4. The consistent failure to select the larger group between 2 and 4 individuals, whether controlled for space occupied or not, suggests that the fish did not only rely on the overall space as 4 fish occupied two times the space. Hence, the authors suggested that zebrafish may use both numerical and non-numerical information to represent quantities (Agrillo et al., 2016; Leibovich et al., 2017; Sheardown et al., 2022) and that attentional constraints and cognitive and working memory mechanisms may orchestrate numerical competence as hypothesized in both humans and other animals (Hyde, 2011). However, these results may also support the hypothesis of distinct quantification systems characterized by domain and task specificity operating largely independently from the others (Feigenson et al., 2004; Miletto Petrazzini et al., 2014).

To date, information on zebrafish’s numerical capacities is scarce and only a few studies used training procedures with stimuli controlled for continuous variables to understand whether zebrafish can use numerical information alone. Agrillo et al. (2012) first compared the numerical abilities of five teleost fishes, (guppies, redtail splitfins, angelfish, Siamese fighting fish, and zebrafish), using the same stimuli, numerical contrasts, and experimental protocol. Fish initially trained to discriminate between two easy numerical contrasts (5 vs 10 and 6 vs, 12; ratio: 0.5) were then tested for their ability to generalize the learned rule both to novel larger numerosities (8 vs. 12 and 9 vs. 12, respectively; ratios: 0.67 and 0.75) and to contrast with constant ratio (0.5), increasing (25 vs. 50) or decreasing (2 vs. 4) total set size. Although only minor differences were observed among the five species, the proportion of zebrafish reaching the learning criterion was lower compared to the other species. They also performed significantly worse in a control experiment testing shape discrimination, suggesting that the observed differences resulted from zebrafish’s difficulty in learning the procedure rather than from a cross-species variation in the numerical domain (Agrillo et al., 2012). However, it has recently been shown that zebrafish trained to discriminate between numbers differing by one unit can successfully distinguish up to 5 vs 6 items (0.83), thus showing excellent learning abilities and numerical skills similar to those observed in higher vertebrates (Bisazza and Santacà, 2022).

Ordinal abilities have also been investigated in zebrafish (Potrich et al., 2019) as previously done in guppies (Miletto Petrazzini et al., 2015). In this study, zebrafish correctly choose the second exit in a series of five identically spaced ones along a corridor based on ordinal information rather than on absolute spatial cues (i.e., total length of the corridor and the distance between inter-exit distance). However, when the number of exits was increased (from 5 to 9) and the inter-exit distance was reduced, they relied both on ordinal and relative spatial information, thus suggesting the use of redundant information to solve a more difficult task (Suanda et al., 2008; Agrillo et al., 2011).

Finally, a habituation/dishabituation paradigm, commonly used to study numerical abilities in newborns (Izard et al., 2009; de Hevia et al., 2014), has recently been adapted to explore the brain regions involved in numerosity discrimination in adult zebrafish through molecular biology analyses (Messina et al., 2020, 2022; see below for more details). In brief, fish were initially habituated to arrays of 3 or 9 dots, changing in item size, position, and density from trial to trial but keeping the same numerousness and overall area. Zebrafish showed a general increase in approach when exposed to a novel stimulus compared to the familiar one even when the stimuli changed in number, indicating that numerousness was being used by the zebrafish.

Several authors suggest the existence of a shared non-verbal numerical system among vertebrates with a possible common genetic basis and evolutionarily conserved neuronal circuits (Agrillo and Bisazza, 2018; Lorenzi et al., 2021). The finding that zebrafish’s abilities to discriminate both continuous and discrete quantities are comparable to those observed in mammals raises the possibility of using this species as a model to investigate both the neural circuitry and the genetic mechanisms underpinning numerosity representation and the role of genes in developmental dyscalculia. Furthermore, some neurodegenerative diseases that have been associated with impaired abilities to estimate both discrete (Gandini et al., 2009) and continuous quantities (Barabassy et al., 2010) could potentially be used for an early diagnosis of disease (Levinoff et al., 2006). The availability of behavioral tools for rapid and easy assessment of quantitative abilities at an early age that can be used for rapid screening of mutant lines for candidate genes potentially associated with such diseases can significantly contribute to progress in biomedical research.

## Neural Circuits for Quantities in Zebrafish: Prey and Predators’ Studies

**FIGURE 1:**
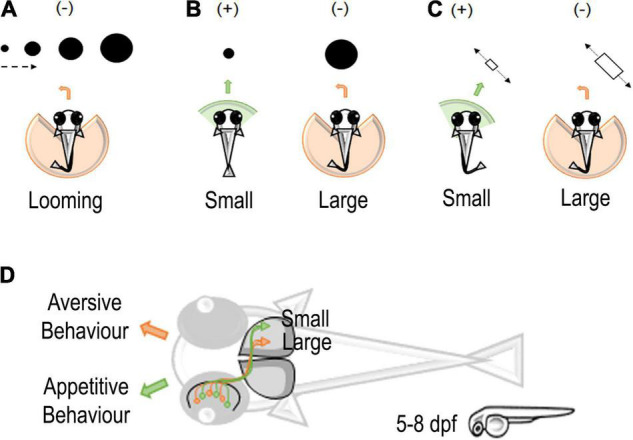
Stimuli and neural circuits associated with prey capture in zebrafish larvae. **(A)** Looming-evoked escape (–) behavior in zebrafish larvae (Temizer et al., 2015). **(B)** A schematic representation of moving small or large dots entering the visual field of zebrafish larvae. Larvae tend to approach (+) small dots and avoid (–) large dots (Barker and Baier, 2015). **(C)** A schematic representation of moving small or large objects in zebrafish larvae. Small objects elicit an approach (+) interaction and large objects an avoidance (–) interaction (Preuss et al., 2014). **(D)** Classification of small and large objects in retinotectal circuits of zebrafish larvae eliciting an appetitive or aversive behavior in zebrafish larvae. Retinal ganglion cells (RGCs) detecting small objects project to the external layers of the zebrafish optic tectum. On the contrary, RGCs for large objects project to the deeper layer of the optic tectum (Preuss et al., 2014).

A key factor for an animal’s survival is prey-predator recognition, an ability that relies on the detection of both the continuous and discrete features of the stimulus (Cross and Jackson, 2017). Size and direction are continuous features and have been well-studied in zebrafish where the location and onset of mediating neurons have been identified (Temizer et al., 2015; Förster et al., 2020). However, the mechanism for detecting discrete quantities is largely unknown and may respond to approximation and/or subitization, namely the instant recognition of the number of objects without sequential counting (Piazza, 2010). Here, we discuss current methodologies and studies on prey-predator behaviors, which is summarized in Figure 1, and how they can be applied to better understand the detection of discrete quantities.

To represent prey and predator interactions, Niell and Smith (2005) used a variety of dot stimulations and found four clusters of tectal neurons that respond to the general movement of a dot, movement in a specific direction, flashing of the dot, or spontaneous darkness (Figure 1A; While these neurons respond intensely to their corresponding categories, they are also triggered by other stimuli. For example, the cluster responding to the general movement of a dot also moderately responds to a flashing, static, or looming dot. Interestingly, the study simultaneously demonstrated the ability to map continuous features to a single neuron using 2-photon calcium imaging. Note, however, that the authors used the calcium indicator dye OGB1 (Oregon Green BAPTA-1), which may have limited their findings due to its reduced signal intensity compared to modern genetically encoded calcium indicators (GECI) as they found large clusters of seemingly unresponsive neurons (Chen et al., 2013). Semmelhack et al. (2014) also found that a small visual area, AF7 (arborization field 7), responds specifically to the optimal artificial prey stimulus based on size and speed (3° dot moving at 90°/s).

Expanding on Niell and Smith (2005) and Preuss et al. (2014) placed a larger emphasis on finding tectal neurons mediating size selectivity. The study used spots ranging from 2° to 64°, incrementing by powers of 2, combined with 2-photon calcium imaging using a GECI. The authors identified that laterally interconnected neurons in the superficial region of the tectum were tuned to smaller (<16°) sizes whereas the deeper tectal region housed neurons tuned to larger (>16°) sizes (Figures 1C,D). These results motivate a question: if a single neuron responds to a single dot, would two visually distinct dots activate two separate tectal neurons? If so, could these two neurons converge to activate a single neuron?

Although neural tuning can demonstrate recognition of a given stimulus, a behavioral output shows that the signal undergoes further processing in other parts of the brain to emit a response. Barker and Baier’s (2015) study found that interneurons in the tectum are required for approach or avoidance during prey-predator interactions (Figure 1B). Typically, zebrafish larvae will approach a moving dot smaller than 5° and avoid dots larger than 10° as early as 5 dpf (Barker and Baier, 2015). However, when a specific glutaminergic tectal neuron was ablated, the authors found an increase in avoidances and a decrease in approaches to small dots. In this study, the behavior was always evaluated using a single object as the stimulus. This leaves an open question: can variation in the number of objects influence approach or avoidance? If given larger groups of small dots, it may be possible for young larvae to approximate or subitize the quantity of a food source as it is evolutionarily advantageous to be efficient foragers.

The discussed studies primarily focused effort on the optic tectum but multiple aspects of the nervous system mediate prey-predator recognition. For example, the visual pathway starts at the retina and primarily connects to the pretectum and the optic tectum, but it then spans to the less explored forebrain and other areas. While this complicates functional studies of the brain, faster and broader imaging techniques are being developed to remedy this. By incorporating proper number-based stimuli with new imaging techniques, it will be possible to characterize the neuronal substrate responsible also for the detection of discrete quantities.

## Neural Circuits for Quantities in Zebrafish: Social Behaviors and the Role of Sensory Features

Despite terrific advancements in linking neural circuits with behavior, thanks to novel methodologies, our knowledge of zebrafish neurobiology exhibit two main gaps (Bollmann, 2019). First, as already discussed, the majority of studies employing functional imaging take advantage of the natural transparency of the skin and the skull of larvae up to around 5 dpf. Therewith, they have concentrated on specific areas that are already mature at that developmental stage (e.g., optic tectum) while neglecting others that go through major structural, as well connectivity changes (e.g., telencephalic areas) (Bloch et al., 2020). The second issue is immediately consequent from the first since the inaccessibility of adult animals to whole-brain functional imaging prevents scientists from investigating some complex behaviors that emerge only at a later age (Valente et al., 2012; Orger and de Polavieja, 2017).

Social behavior is one example, given that zebrafish juveniles start to approach groups of conspecifics at 7 dpf and completely mature social behaviors only at 21 dpf (Dreosti et al., 2015). The growth of social drive seems to follow an exponential growth between 6 and 24 dpf (Hinz and de Polavieja, 2017), which parallels the maturation of the nervous system. During this period, they start to display orientation toward other fish, shoaling, schooling, and a greater preference for more numerous groups of conspecifics as previously mentioned.

Besides the development of social drive, one central question regarding these behaviors is what triggers them. The necessity to study social behavior in dynamic contexts has prevented investigating the role of sensory signals and perceptual processes. However, understanding the mechanisms and neural circuits involved is a primary issue.

Visual cues are necessary for social behaviors since the removal of illumination or the occlusion of visual stimuli removed any social preference, orientation, or shoaling behavior in fish (Dreosti et al., 2015; Larsch and Baier, 2018; Harpaz et al., 2021). We know that several physical factors influence social behaviors, such as stimulus size, shape, motion, and color (Abaid et al., 2012; Gerlai, 2017). Also, the number of stimuli affects social preference and decision-making (Pritchard et al., 2001; Arganda et al., 2012).

Interestingly, computer-generated dots exhibiting age-specific characteristic swim kinetics can elicit shoaling in juvenile (15–27 dpf) or adult zebrafish (Larsch and Baier, 2018). Reciprocal interaction is not necessary to start shoaling, while attraction was strongest to dots of 1.8–3.7 mm. This preference mirrors the actual size of body parts more salient for fish (e.g., head, torso). Consistently, older animals tend to prefer larger dots.

**FIGURE 2:**
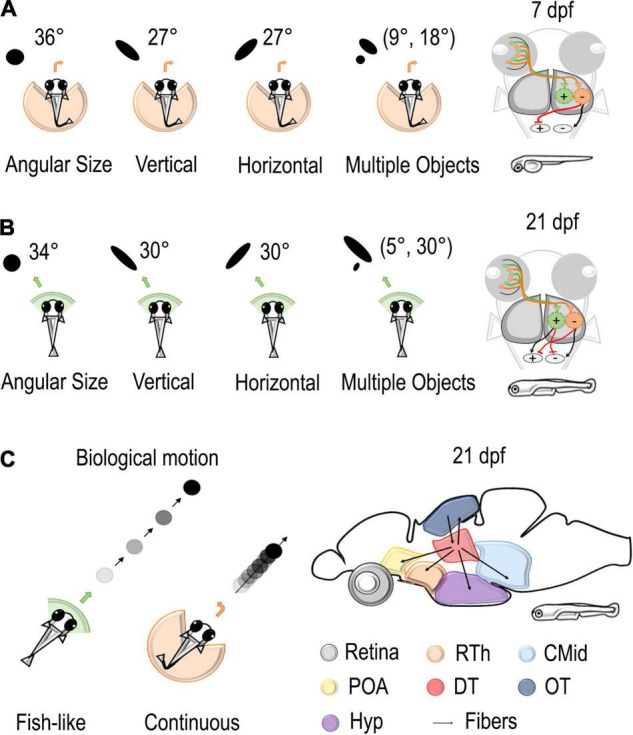
Stimuli and neural circuits involved in social behaviors. **(A,B)** *Left*: Several types of stimuli were presented to the visual field of zebrafish using a virtual reality assay. Black dots vary in angular size, vertical dimension, horizontal dimension, and number. At 7 dpf, these stimuli elicited an aversive turn with an increasing probability of repulsion with an increase in the retina occupancy. At 21 dpf, stimuli of the same size were considered attractive. *Right*: the model proposed by Harpaz et al. (2021) hypothesized the existence of two different populations in downstream areas that are responsible for attraction or avoidance of social stimuli. While only the repulsive population is mature at 7 dpf, the balance of excitation and inhibition of the two populations determines the behavior of the fish at 14–21 dpf. **(C)** *Left*: black dots with bout-like motion elicited shoaling behavior in juvenile zebrafish, while continuously moving dots were considered not attractive (Larsch and Baier, 2018). *Right*: Connectivity patterns of dorsal thalamus where Kappel et al. (2021) found a cluster of neurons selective for bout swim. Rth, rostral thalamus; POA, preoptic area; CMid, caudal midbrain; DT, dorsal thalamus; OT, optic tectum; Hyp, hypothalamus.

A specific visual circuit involved in the selection of larvae characteristic swim kinetics (bout-like acceleration) was found in juveniles of 21 dpf (Kappel et al., 2021, see Figure 2). Global activity maps by *c-fos in situ* hybridization chain reaction (HRC) revealed a cluster of brain areas whose activity was modulated by virtual and real conspecifics. These involve some evolutionary conserved hypothalamic components and the optic tectum, the dorsal thalamus (DT), and the posterior tuberculum, hinting at interesting roles in the visual pathway. Two-photon calcium imaging confirmed a posterior cluster of neurons in the dorsal thalamus selective for bout swim (bout preference neurons, BPN). Interestingly, this activation was specific for acceleration at 5 mm/s fish-like speed and characteristic bout frequency. These neurons are already present in larvae although at a lower number. Analysis of connectivity by electron microscopy revealed not only projections from the tectal periventricular neurons to the dorsal thalamus but also connections of these back to the tectum or directed to the hypothalamus. A possibility is that during development DT neurons mature projections to hypothalamic as well forebrain areas, such as the ventral forebrain that are known to be causally involved in social behavior in adults (Stednitz et al., 2018; Tallafuss et al., 2022). Chemogenetic ablation of tectal cells reduced the number of BPN active neurons in the dorsal thalamus by more than 80% and determined loss of attraction toward moving dots in 21 dpf juveniles. Despite the disruption of shoaling and inversion in the characteristic ring of attraction, collision avoidance and escape responses to looming stimuli were intact, thus suggesting diversification of circuits for these behaviors.

Retinal occupancy and physical features, such as area, shape, and number of stimuli, are particularly relevant for collective behaviors. Thanks to virtual reality assay, Harpaz et al. (2021) were able to study their role in moving stimuli (black dots) projected to the retinal space by varying each variable at a time while keeping all the others constant (Figure 2). They could notice that, at 7 dpf, an increment in the angular size increases the probability of an aversive turn, while, at 21 dpf, dots as large as 45° were considered attractive social stimuli. The vertical size of the stimuli had a greater relevance for animals. Changing the height of the dot increased escape behavior in 7–21 dpf, but an increase in width did not have an effect on 7 dpf fish and a moderate one in 14–21 dpf fish. Concerning the number of dots, animals seemed to compute a weighted average of the response to each stimulus presented alone, with weight proportional to its size. Thus, visual occupancy seems primary concerning the number and density of stimuli.

The consistent role of retinal occupancy but the different behavioral responses at 7, 14, and 21 dpf made Harpaz et al. (2021) hypothesize the existence of two different populations in downstream areas that are responsible for attraction or avoidance toward stimuli (Figure 2). According to this model, retinal ganglion cells map the vertical height of stimuli at each visual angle and project to two different populations of neurons: repulsive and attractive. There, RGC activity is first integrated and later averaged by specific units. These output units then send excitatory/inhibitory projections to motor centers determining the turning direction of the fish. While only the repulsive population is mature at 7 dpf, the balance of the two populations determines the behavior of the fish at 14–21 dpf. According to this model, the quantity estimation process is already mature at 7 dpf, while the control of behavior changes over development.

Moving to other sensory modalities, mechanosensitive neuromast cells of the lateral line also contribute to the perception of conspecifics, modulating the expression of parathyroid hormone 2 (pth2) of the dorsal thalamus neurons in zebrafish (Anneser et al., 2020). Interestingly, Pth2 expression follows a quantitative relationship with the social environment of the animals throughout all developmental stages (5–21 dpf) and adulthood. Indeed, while isolated fish showed a rapid reduction of the level of pth2, its expression linearly increased with the number of fish present. Also, pth2 seems sensitive to the current social setting and its expression is not altered in the long term. However, when visually stimulated while physically isolated by a glass barrier, pth2 levels were not altered. Therefore, pth2 expression seems specific for mechanical stimulation and strongly tracks the group size of conspecifics. Remarkably, these pth2 expressing neurons overlap with the bout preference cells in the dorsal thalamus (Kappel et al., 2021). This might suggest the dorsal thalamus as a possible multisensory area integrating sensory signals of conspecifics. Recently, a loss-of-function mutation in the gene pth2 lowered shoal cohesion in adults (Anneser et al., 2022), suggesting a role of this hormone in regulating downstream signals important for shoaling.

## Neural Correlates of Continuous and Discrete Magnitude in Zebrafish Adult Brain

**FIGURE 3:**
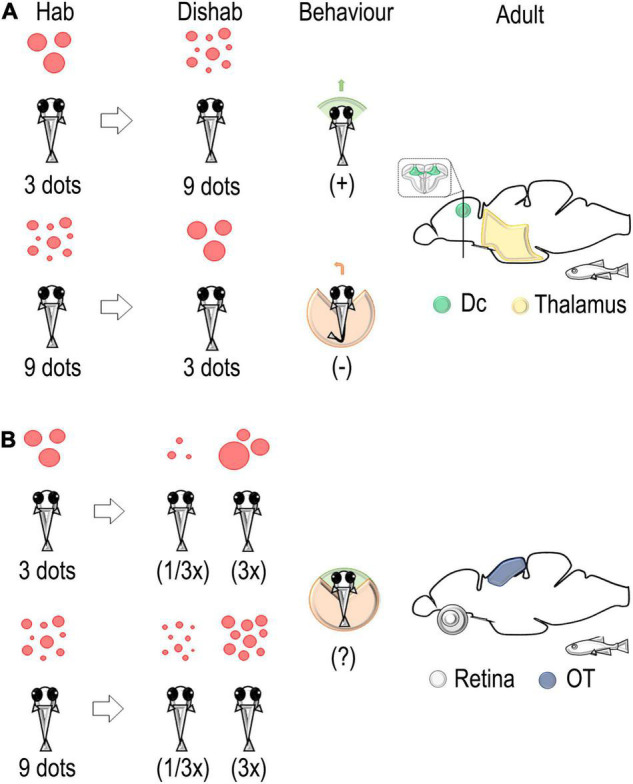
Schematic representation of the habituation/dishabituation paradigm used to identify neural correlates associated with a change in discrete and continuous magnitude in the adult zebrafish brain. **(A)** A change in number from 3 (habituation) to 9 (dishabituation) dots elicits an approach interaction. On the contrary, avoidance of the stimulus is detected in the change from 9 to 3 dots. An evaluation of immediate early genes (IEG) expressions revealed the main role of the thalamus and the dorso-central pallium (Dc) in the elaboration of changes in discrete quantity (numerosity) (Messina et al., 2020, 2022). **(B)** A change in size, i.e., a decrease (1/3x) or an increase (3x), revealed the main role of the retina and the optic tectum in the elaboration of continuous quantity (stimulus size) (Messina et al., 2020, 2022).

Recently, part of the neural network associated with the estimation of continuous and discrete quantity in the zebrafish brain has been identified (Messina et al., 2020, 2022). Adult zebrafish were trained to familiarize themselves (habituation phase) with artificial stimuli, small red dots (three or nine dots) that changed in individual position, density, and size, while maintaining their numerousness and overall surface from trial to trial. During the subsequent dishabituation phase, separate groups of fish faced different types of change in the stimuli: a change in number (nine or three dots with the same overall surface); in size (with the same shape and number); in shape (squares instead of dots); and no change at all (control group). Combining this spontaneous habituation/dishabituation paradigm with the evaluation of the expression of specific markers of neural activity, i.e. the immediate early genes *c-fos* and *egr-1* (IEGs), we found a specific activation of the thalamus and the most caudal part of the dorso-central (Dc) pallium in fish facing a change in numerosity (Figure 3A) concerning the other experimental conditions (Messina et al., 2020, 2022). These results agree with evidence reporting the involvement of thalamic nuclei in 10-year-old children performing quantity estimation in fMRI studies (Kovas et al., 2009) and with the relevant role of subcortical (and more generally subpallial) territories in tasks involving magnitude estimation (review in Lorenzi et al., 2021; Vallortigara et al., 2022). Also, they are in line with the response to numerosity shown by single-cell recording experiments in the parietal and prefrontal cortex in the non-human primate cortex (Nieder et al., 2002; Nieder and Merten, 2007; Nieder, 2016; Viswanathan and Nieder, 2020), in the nidopallium caudolateral of crows (Ditz and Nieder, 2015, 2016) and young chicks (Kobylkov et al., 2022), and in fMRI experiments in humans (Piazza et al., 2004). Further research will be needed to clarify the possible homologies/analogies between the dorso-central pallium (Dc) of zebrafish and the cortical/pallial areas responding to numerosity in the mammalian and avian brains (Nieder, 2021b).

Hodological studies suggest that Dc contains the major descending pathway of the fish pallium to the optic tectum and medulla oblongata (Yamamoto and Ito, 2005; Yamamoto et al., 2007; Ito and Yamamoto, 2009; Harvey-Girard et al., 2012; Rodríguez et al., 2021). Intriguingly, we found that the amount of *egr-1*-positive cells in Dc tended to increase with changes from small to large numerosity and to decrease with changes from a large to a small set of dots, suggesting that a higher or lower activation of Dc could be related to a higher/lower response of fish motor responses (approach or avoidance) in association with the direction of the change in numerosity; this was also supported by different behavioral measures (Messina et al., 2022).

As to the response to continuous quantity as opposed to discrete ones (Figure 3B), we found a selective modulation in the expression levels of IEGs in the retina and the optic tectum in the groups of zebrafish facing a change in the size of the stimulus (a three-fold increase or decrease in the size of the individual dots; see Messina et al., 2020). These results support previous evidence in zebrafish larvae that showed an involvement of the optic tectum in the categorization of visual targets of different sizes in approach/avoidance behavior (Abbas and Meyer, 2014; Preuss et al., 2014). Furthermore, stimulus-size selectivity was also reported in studies of the intratectal circuitry involved in the prey-catching behavior of amphibians (Ewert and Gebauer, 1973; Cervantes-Pérez et al., 1985; Ewert, 1987) and in single cells recording experiments in pigeons (Gusel’nikov et al., 1971), suggesting a general mechanism of activation of tectal circuits about continuous magnitude discrimination in vertebrates (see also Lorenzi et al., 2021).

Further research is ongoing to detail the ascending and descending pathways driving the response to continuous and discrete magnitude-related visual stimuli from the retina, through the thalamic and optic tectum nuclei, toward the dorsal pallium and the premotor and motor nuclei of the zebrafish brain.

## Modeling Developmental Dyscalculia in Fish

As discussed above, the finding that zebrafish’s abilities to discriminate both continuous and discrete quantities are comparable to those observed in mammals raises the possibility of using this species as a model to investigate both the neural circuitry and the genetic mechanisms underpinning numerosity representation and the role of genes in developmental dyscalculia (DD). DD is a recently recognized congenital condition that results in an impaired ability to perform simple arithmetic operations. It describes children who fail to achieve adequate arithmetic proficiency despite normal intelligence, scholastic opportunity, emotional stability, and necessary motivation (APA, 2013). The disorder has a high prevalence of 5–7% (Shalev, 2007; von Aster and Shalev, 2007). Dyscalculic individuals are found to be unable to grasp the abstract concept of number (cardinality) by the expected age, meaning that they will also be unable to learn the place principle (ordinality) and calculation (Kaufmann et al., 2013; Rubinsten, 2015). These learning difficulties have lifelong consequences, correlating with socioeconomic status (Ritchie and Bates, 2013). Deficits in symbolic mathematics have also been found to be associated with low performance in non-symbolic numerical tasks (Piazza et al., 2010; Mazzocco et al., 2011).

Like other learning disabilities, DD has a significant familial aggregation (Shalev et al., 2001) with a strong effect of genetic influence (Alarcón et al., 1997; Oliver et al., 2004; Kovas et al., 2007). This, along with the clinical characterization of dyscalculia as a syndrome, led to Genome-Wide Association Studies (GWAS) being undertaken. Docherty et al. (2010) used a twin study to investigate the genes affecting mathematical ability and disability. Twins in England and Wales were given web-based testing and the National Curriculum Review ratings, which were combined to give a composite measure of mathematical ability. Stage 1 used the 16th percentile of the top and bottom performers in the composite score, with 300 subjects for extremely high and extremely low performance. Stage 2 took the 20th percentile and stage 3 used individuals with a range of abilities. GWAS looking for Single Nucleotide Polymorphisms (SNPs) associated with mathematical performance in stage 1 found 46 candidate SNPs. These results were validated in stage 3, where 10 SNPs remained significantly associated with individual differences in mathematical ability. The nearest genes to these SNPs were MMP7, GRIK1, DNAH5, SMAD3, ARID1B, FLJ20160, GUCY1A2, NRCAM, DLD, and NUAK1, making them potential candidates for influencing numerical processing. No overall large effect was found in the study suggesting the genetic influence on mathematical ability is caused by multiple Quantitative Trait Loci (QTL) of small effect (Docherty et al., 2010). A more recent GWAS study was performed by Chen and colleagues. The mathematical abilities of school-age children were assessed by looking at the midterm and final math exam results in each semester. A combined meta-analysis identified four SNPs associated with mathematics ability. All these SNPs were located on the SPOCK1 gene, a gene previously implicated in neurodevelopment through neurogenesis, now a potential candidate for the development of mathematical ability (Chen et al., 2017).

Other genetic studies have identified candidates for developmental dyscalculia. Quantitative data on mathematic ability was correlated with genome data of 200 children with dyslexia and found that the rs133885 variant in the myosin-18B (MYO18B) gene is the only marker that had an association with the mathematical ability at a statistically significant level. Neuroimaging of 79 healthy adults showed that carriers of the rs133885 risk allele displayed a reduced depth of the right intraparietal sulcus, which has been proposed to mediate numerical processing (Ludwig et al., 2013). A Copy Number Variant (CNV) scan for psychiatric conditions in the Icelandic population also identified a region in chromosome 15q11.2 between breakpoints 1 and 2 [15q11.2 (BP1-BP2) deletion] in controls with a history of dyslexia and dyscalculia, disrupting GCP5, CYFIP1, NIPA2, and NIPA1 (Stefansson et al., 2014; Ulfarsson et al., 2017). This region is a recurrent site for chromosomal rearrangements underlying different neurodevelopmental conditions.

Another way to identify DD-associated genes is to look at syndromes with a dyscalculia component and any genes associated with those syndromes. For example, Fragile X syndrome (FXS) and Prader–Willi Syndrome (PWS) are human genetic disorders that are associated with poor number cognition. FXS is the most common cause of inherited intellectual disabilities. It is caused by the expansion of trinucleotide CGG in the fragile X mental retardation 1 gene (FMR1) (Bagni et al., 2012). Additionally, premutation expansions (55–200 repeats) can cause neurodevelopmental problems such as deficits in numerical cognition and arithmetic (Murphy et al., 2006; Sullivan et al., 2006; Cordeiro et al., 2011; Goodrich-Hunsaker et al., 2011; Lee et al., 2012; Tassone et al., 2014). CYFIP1 Interacts with FMR1 as part of the CYFIP1-FMR1-eIF4E pathway. This pathway affects synaptic plasticity through its role as a negative regulator of translation. FMR1 binds to CYFIP1, and the FMR1-CYFIP1 complex binds to the translation initiation factor eIF4E. This inhibits the eIF4E-mediated initiation of translation, thereby affecting the translation of a large group of target messenger RNAs (mRNAs), particularly those found at synapses. Known targets include ARC (AKA Arg3.1), MAP1B, CAMKII, PSD-95, GLUR1, and GLUR2 (Abekhoukh et al., 2017).

In PWS, although there is a very severe phenotype, mathematical abilities are more impaired relative to other cognitive functions and approximately 70% of PWS cases are caused by the deletion of 15q11-13. As mentioned above, CYFIP1 has been found to be one of the 4 genes in the genes in this 15q11.2 copy number variation, which when deleted confers for risk of dyscalculia (Bertella et al., 2005). The Cytoplasmic FMR1-Interacting Protein (CYFIP) gene is highly conserved and expressed in the central nervous system. CYFIP1 and CYFIP2 are enriched at inhibitory synapses and have a role in regulating the balance between excitatory and inhibitory synapses (Davenport et al., 2019). CYFIP1 and CYFIP2 also consistently appear in the top 10% of hits in published GWAS studies (Davis et al., 2014; Chen et al., 2017), suggesting that they may play a role in developmental dyscalculia.

In addition to FXS and PWS, individuals with William’s syndrome (WS) have been found to show dyscalculia phenotypes with a range of deficits and impairments in aspects of their numerical abilities (Ansari et al., 2007; O’Hearn et al., 2011; Opfer and Martens, 2012). Deficits in elements of patients with WS’ symbolic (Krajcsi et al., 2009) and non-symbolic (Rousselle et al., 2013) systems have been observed. Another study found the non-symbolic system (specifically the ANS) of WS adolescents is functioning at the same level as 2–4-year-old Typical development (TD) children, whereas the symbolic system is comparatively functioning at a much similar level to TD 6–9-year-olds. This suggests that WS individuals have greater impairment in their non-symbolic abilities in comparison to their symbolic abilities (Libertus et al., 2014).

William’s syndrome (WS) is genetically defined by a typical hemizygous 7q11.23 microdeletion of 1.55 million base pairs (Mb) that encompasses approximately 28 genes (Bayés et al., 2003). One of the genes in this microdeletion is BAZ1B (bromodomain adjacent to zinc finger domain, 1B), also known as Williams syndrome transcription factor (WSTF) (Kitagawa et al., 2011). BAZ1B is an evolutionarily conserved protein tyrosine kinase and is expressed throughout neurodevelopment. WS neurons show defects in differentiation influenced by haploinsufficiency of BAZ1B with widespread gene expression changes in neural progenitor cells (Lalli et al., 2016). This haplosufficiency explains 42% of the transcriptional dysregulation seen in WS neurons. Another gene in the WS microdeletion is FZD9 (Frizzled class receptor 9) (Kitagawa et al., 2011). Frizzled receptors are the mediators of the WNT pathways, which play fundamental roles in cell differentiation and organism development (Corda and Sala, 2017). The study of patients with WS found that their neurons had longer dendrites, an increased number of spines along with aberrant calcium oscillations, and altered network connectivity, which were found to be caused by FZD9 (Chailangkarn et al., 2016). Their role in neuron dysfunction in WS highlights these two genes as potential candidates for a causal role in developmental dyscalculia.

Neural networks are highly conserved across vertebrates, and zebrafish have a relatively simple neural system and high homology with the human genome (Howe et al., 2013), supporting the translational validity of the model for functional validation. The establishment and subsequent optimization of genome editing technologies in zebrafish have enabled reverse genetic approaches to be used in the model for testing the functional role of GWAS-associated loci using the highly efficient CRISPR/Cas9 system (Hwang et al., 2013). Reverse genetic approaches are used to test the hypotheses regarding a causal role, a developmental role, or a mechanism of action of a candidate gene associated with human disorders. These have been used to great effect in zebrafish as endophenotypes of disorders can be tested in robust assays that, coupled with CRISPR/Cas9 loss of function and molecular assays, can define causal genes involved in the disorders. A gene expression screening of nine genes (baz1b, fzd9, limk1, tubgcp5, cyfip1, grik1a, robo1, nipa1, and nipa2) is associated with human developmental dyscalculia (Chai et al., 2003; Tassabehji, 2003; Docherty et al., 2010; Maver et al., 2019), which revealed that most of them are largely distributed in the zebrafish dorsal pallium and five of them (grik1a, nipa1, nipa2, and robo1) are asymmetrically distributed between the left and the right hemispheres, opening the way to another crucial theme that links developmental dyscalculia with brain laterality (Shalev et al., 1995; Shalev, 2004; Messina et al., 2021a). With the establishment of robust assays of numerical abilities in zebrafish, we are well placed to test the causal roles of candidate genes and their mode of action.

## Future Directions: New Methodologies in Calcium Imaging for Next-Gen Studies

Recent advancements in live imaging have enabled recording the functional activity map of large areas, and entire volumes, of the brain of small model organisms (e.g., *C. elegans*, drosophila, and zebrafish), while the animals undergo naturalistic behavior. This brain-wide imaging approach enables the exciting prospect of unbiased observation and identification of the relevant brain substrates that underlie behavior. A comprehensive review of these imaging advancements is beyond the scope of this contribution and has been done elsewhere (Weisenburger and Vaziri, 2018). Here, we focus on the discussion of key developments that could provide relevant advancements for combining imaging of zebrafish neural circuits with behavior. In particular, given the lack of live imaging studies investigating the detection of discrete quantities in comparison with the copious literature on size detection, we suggest an application of these methods for the study of numerosity in zebrafish.

As discussed previously, most zebrafish numerosity or numerosity-related studies have involved the animal’s visual response to numerosity stimuli. Thus, when imaging this numerosity response, it is critical to ensure that the animal’s visual response is not compromised by the laser light used to excite the fluorescence signals. Zebrafish can detect wavelengths in the visible range of ∼400–650 nm (Cameron, 2002), hence ruling out the usage of conventional 1-photon excitation with visible wavelengths. Thus, to avoid interfering with the zebrafish’s visual response, frequent imaging should be carried out with 2-photon excitation, where the pulsed laser light has wavelengths in the near-infrared range, ∼800–1,000 nm, which is invisible to the zebrafish. We will, thus, focus our discussion below mainly on imaging technologies that use 2-photon excitation.

A mainstream of live neuroimaging is the modality of 2-photon excitation point-scanning microscopy (2p-PSM) (Denk et al., 1990), where the 3D volume of interest is imaged one voxel at a time by serially raster-scanning the point over the volume. Recent refinements in better 2-photon laser sources, better photon-counting detectors, and better design of optical detection pathways to maximize the photon collection efficiency, and a plethora of turn-key user-friendly commercially-available instruments at affordable prices (Weisenburger and Vaziri, 2018; Bruzzone et al., 2021), have increasingly made 2p-PSM available to the zebrafish neuroscience community. In 2p-PSM, the achievable 2D (frame) imaging rate is typical ∼1 Hz with systems that use a galvanometer scanner and ∼30 Hz with systems that use a resonant scanner. Volume coverage is achieved by scanning the imaging frame axially, thus achieving a volumetric imaging rate that scales inversely with the number of z-slices (e.g., 10 z-slices recorded at 1 Hz frame rate will yield ∼0.1 Hz volumetric rate).

In the effort to improve the imaging speed of 2p-PSM and reduce the potential photodamage associated with the intense peak laser intensity needed for the point-scanning and serially-recorded strategies, new parallelized-recorded imaging modalities have been developed. In 2-photon light-sheet microscopy (2p-SPIM) (Truong et al., 2011), the excitation is achieved by scanning a gently-focused pencil-like laser beam to create the sheet-like fluorescence signal along the detection focal plane, which is then imaged by a camera. The parallelized 2D image collection enables a longer signal integration time for each voxel, thus requiring a much-reduced peak laser intensity compared with 2p-PSM. Recent implementations of 2p-SPIM, as applied to brain-wide functional imaging of zebrafish (Wolf et al., 2015, 2017; Keomanee-Dizon et al., 2020; de Vito et al., 2022), have achieved imaging frame rates of 20–150 Hz and volumetric rates spanning ∼1–5 Hz. Importantly, in these studies, the upper limit of the imaging rate is not dictated by the imaging hardware but rather by the putative threshold of photodamage, which starts to appear as the laser power is increased.

Taking the parallelized-recording strategy to the third dimension, light field microscopy (LFM) is an imaging modality that uses a plenoptic (i.e., multi-view) detection strategy to capture an entire 3D volume of interest in a single 2D camera snapshot and computation to reconstruct the original image volume (Levoy et al., 2006). Thus, a typical imaging frame rate of ∼30 Hz will readily yield a very fast volumetric rate of 30 Hz after reconstruction. While the resolution achieved is necessarily reduced to provide the extended volume coverage beyond the native focal plane (as compared to conventional modalities that record only the focal plane), light-field-based imaging approaches still achieve single-neuron resolution over depths of 100 microns or more in imaging the zebrafish brain (Prevedel et al., 2014; Cong et al., 2017; Truong et al., 2020). The latest developments and applications of LFM in neuroimaging of zebrafish (Lin et al., 2020; Zhang et al., 2021) showed great promise to provide fast, volumetric imaging of multiple-regions or whole-brain coverage of zebrafish during naturalistic behavior. Light-field-based selective volume illumination microscopy (SVIM) (Truong et al., 2020; Madaan et al., 2021) combines the spatially-selective illumination strategy of light-sheet microscopy with light-field detection to significantly improve the signal contrast while maintaining the high volumetric imaging rate of LFM. SVIM has also been implemented with 2-photon excitation, providing a promising way to study visually-sensitive processes, such as numerosity in zebrafish. One general challenge with light-field-based techniques is the substantial amount of time and computational power needed to carry out the image reconstruction post-acquisition, which typically requires ∼10^3^ times longer to reconstruct compared to the imaging volumetric rate. Toward mitigating this computation challenge, a variant of LFM called Fourier-LFM (Cong et al., 2017; Guo et al., 2019), by operating with a spatially-invariant point-spread-function, reduces the reconstruction time by ∼2 orders of magnitude, thus bringing LFM closer to the wide-spread application to neuroscience research.

The above-described trends in the maturation/development of established/novel imaging technologies have enabled exciting progress in zebrafish neuroscience in recent years. We highlight here several of these works. In Lee et al. (2017), 2p-SPIM was used to image 6-dpf zebrafish undergoing sleep and wake, to monitor the brain-wide activity map as a function of natural and optogenetically driven sleep. Of note, the 2p-SPIM imaging was gentle and visually inert, enough to not disrupt the normal sleep/wake behavior of the zebrafish. Wagle et al. (2022) used 2p-PSM with 5–7-dpf zebrafish to study the brain-wide perception of the emotional valence of light and found that it is regulated by the distinct hypothalamic corticotropin-releasing factor neurons. Here, again, the visual inertness of the 2-photon excitation light was critical to enable brain-wide imaging while the animals were treated to cycles of light/dark stimuli. Finally, 2p-SPIM was used by de Vito et al. (2022) to map the whole-brain activity patterns of 4-dpf zebrafish undergoing epileptic seizures. The authors characterized the spatial-temporal dynamics of the pathological seizure activity and identified a previously-unknown caudo-rostral ictal wave pattern.

**FIGURE 4:**
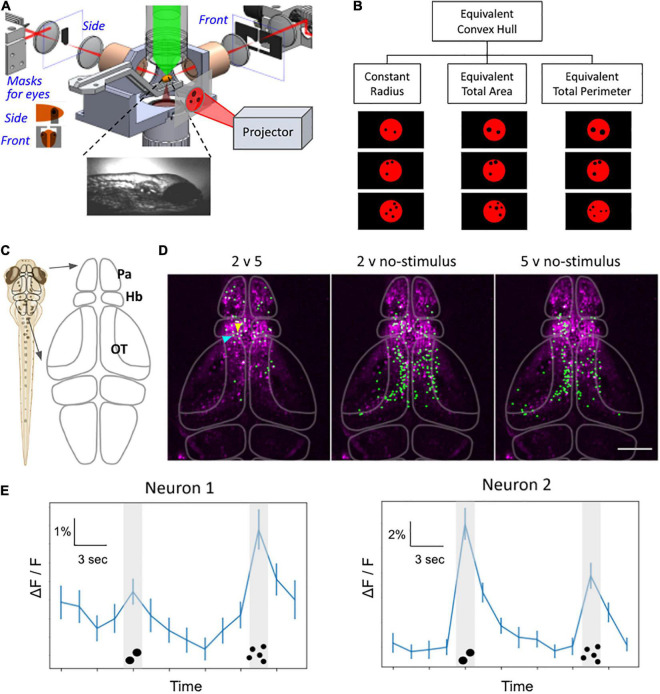
Whole-brain functional imaging to find neural substrate of zebrafish numerosity capability. **(A)** Schematic setup of the 2p-SPIM setup that allows calcium imaging of awake zebrafish larvae that are subjected to visual numerosity stimuli. **(B)** Examples of the numerosity stimuli, which consist of a particular number of black dots on a diffuse red background. The visual patterns are controlled for various non-numerosity continuous variables to find the responses that are intrinsic to the recognition of discrete numbers. **(C)** Schematic drawing of the brain. The forebrain includes the pallium (Pa) and the habenula (Hb). The midbrain contains the optic tectum (OT). **(D)** Representative results from 8-dpf larvae show neurons that exhibited different levels of activity during different stimuli (e.g., 2 versus 5 dots, etc.). Green: nuclei of identified neurons. Magenta: anatomical background generated via the maximum-intensity projection in Z of the standard deviation projection in time, depicting neurons with time-varying activity. The raw dataset covers the volume of ∼350 μm × 600 μm × 250 μm (depth), taken with three sections per volume for 42 min. **(E)** Representative activity traces, of the two neurons selected by arrowheads in panel **(D)**, during number stimuli. The line graph depicts activity levels as ΔF/F for the time trial as shown, averaged over *n* = 50 trials. Neuron 1 (blue arrowhead): higher activity levels during the 5-dot stimuli compared to the 2-dot stimuli. Neuron 2 (yellow arrowhead): higher activity levels during the 2-dot stimuli compared to the 5-dot stimuli. *P* < 0.05, bootstrapping with resampling. Error bars represent SEM, *n* = 50. Scale bar in panel **(D)**,100 μm.

Together, the studies described above point to the exciting possibility that the numerosity capability of zebrafish could be studied using brain-wide functional imaging to identify not only its neural substrate (i.e., “number cells”) but possibly also the circuits involved in how the animal processes number stimuli. Toward this goal, we have started a research program employing whole-brain functional imaging to study zebrafish numerosity. We highlight some of our preliminary results in Figure 4. We used the microscopy platform described in Keomanee-Dizon et al. (2020) to record the whole-brain activity map of awake zebrafish larvae, which were transgenically labeled with fluorescent calcium indicators. A projector system was used to present visual numerosity stimuli to the zebrafish (Figure 4A). The stimuli consisted of a particular number of black dots on a diffuse red background, and the repeated presentations were controlled to find the activity patterns that are intrinsically sensitive to the number of stimuli and not to other continuous variables of the dot patterns (such as radius, total area, total perimeter, etc.) (Figure 4B). In an exemplary recording, the fish were presented with multiple trials of blank (no stimulus), 2 dots, blank, and then 5 dots, as depicted in Figure 4E. We then used a T-score-based analysis approach to identify neurons that exhibited a difference in their activity under the different stimuli (Figure 4D). We found that many neurons have differential activity between 2 dots (or 5 dots) versus blank, particularly in the optic tectum. Promisingly, we also found neurons, located mainly in the pallium and the habenula (Figure 4D), that have differential activity between 2 vs 5 dots. The forebrain location for these putative number neurons is consistent with our team’s results in adults results (Messina et al., 2020, 2022), discussed earlier in Section “Neural Correlates of Continuous and Discrete Magnitude in Zebrafish Adult Brain”. We are poised to confirm and expand the preliminary results shown here to fully characterize the cellular substrate of the numerosity capability of the zebrafish larvae.

## Conclusion

The main goal of this review was to provide a state of the art of our current behavioral and neurobiological understanding of the mechanisms underlying quantity (discrete and continuous) encoding and processing in fish.

Although the ability to estimate quantities was documented in both invertebrates and vertebrates and the involvement of subpallial and pallial brain structures was described in different taxonomic groups in vertebrates (mammals, birds, and fish; reviews in Lorenzi et al., 2021; Messina et al., 2021b; Nieder, 2021b; Vallortigara et al., 2022), the exact neural circuits devoted to the elaboration of continuous and discrete magnitudes have not been precisely identified as of yet.

Zebrafish represents an excellent animal model system to investigate the genetics and molecular mechanisms of behavior and may be instrumental to develop the neurobiology of magnitude cognition with a complete characterization of neurons and neural circuits associated with quantity discrimination processes, laying the foundations for comparative molecular studies in other animal species, including humans.

## Author Contributions

AM, DP, MP, ES, MM, PL, AN, TT, VS, SF, CB, and GV conceived the manuscript and performed the literature search. SF, CB, and GV reviewed the manuscript and contributed to financial support. AM, DP, MP, ES, MM, PL, and TT contributed to the writing—original draft. All authors edited and then approved the submitted version.

## Conflict of Interest

The authors declare that the research was conducted in the absence of any commercial or financial relationships that could be construed as a potential conflict of interest.

## Publisher’s Note

All claims expressed in this article are solely those of the authors and do not necessarily represent those of their affiliated organizations, or those of the publisher, the editors and the reviewers. Any product that may be evaluated in this article, or claim that may be made by its manufacturer, is not guaranteed or endorsed by the publisher.
